# Morbillivirus and coronavirus survey in stranded cetaceans, Brazil

**DOI:** 10.1371/journal.pone.0316050

**Published:** 2025-03-10

**Authors:** Samira Costa-Silva, Carlos Sacristán, Arícia Duarte-Benvenuto, Ana Carolina Ewbank, Rodrigo M. Soares, Vitor L. Carvalho, Pedro V. Castilho, Marta J. Cremer, Jenyffer V. Vieira, Giulia G. Lemos, Jéssica R. Moreira, Gladys D. Rogge Renner, Cristiane K. M. Kolesnikovas, Natalia S. Peres, Thalita Faita, Larissa Pavaneli, Joana Ikeda, Adriana C. Colosio, Milton C. C. Marcondes, Angélica M. Sánchez-Sarmiento, Carla B. Barbosa, Raquel B. Ferioli, Vanessa L. Ribeiro, Carolina P. Bertozzi, Caroline F. Pessi, Henrique Chupill, José L. Catão-Dias, Lara B. Keid

**Affiliations:** 1 Faculdade de Medicina Veterinária e Zootecnia – Universidade de São Paulo, São Paulo, Brazil; 2 Centro de Investigación en Sanidad Animal (CISA-INIA), CSIC, Valdeolmos, Madrid, Spain; 3 Associação de Pesquisa e Preservação de Ecossistemas Aquáticos - AQUASIS, Caucaia, Ceará, Brazil; 4 Universidade do Estado de Santa Catarina-UDESC, Laguna, Snata Catarina, Brazil; 5 Laboratório de Ecologia e Conservação de Tetrápodes Marinhos e Costeiros - TetraMar, Universidade da Região de Joinville, São Francisco do Sul, Santa Catarina, Brazil; 6 Laboratório de Processamento Histológico – LAPHIS, Universidade da Região de Joinville, São Francisco do Sul, Santa Catarina, Brazil; 7 Associação R3 Animal, Florianópolis, Santa Catarina, Brazil; 8 Instituto Mamíferos Aquáticos - IMA, Salvador, Bahia, Brazil; 9 Instituto Baleia Jubarte – IBJ, Caravelas, Bahia, Brazil; 10 Instituto Argonauta para a Conservação Costeira e Marinha, Ubatuba, São Paulo, Brazil; 11 Instituto Biopesca, Praia Grande, Santa Catarina, Brazil; 12 Instituto de Biociências, Universidade Estadual Paulista (UNESP), São Vicente, São Paulo, Brazil; 13 Instituto de Pesquisas de Cananéia, Cananéia, São Paulo, Brazil; 14 Faculdade de Engenharia de Alimento e Zootecnia – Universidade de São Paulo, Pirassununga, São Paulo, Brazil; Sudan University of Science and Technology, College of Veterinary Medicine, SUDAN

## Abstract

Since 2010, Guiana dolphin morbillivirus (GDMV; family *Paramyxoviridae*, genus *Morbillivirus*, species *Morbillivirus ceti*, syn. *Cetacean morbillivirus*) is recognized as the cause of death of multiple cetacean species along the Brazilian coast, including an unusual mortality event in Rio de Janeiro state. Coronaviruses of the genus *Gammacoronavirus* (family *Coronaviridae*) have been previously detected in cetaceans in the northern hemisphere. After the emergence of the severe acute respiratory syndrome coronavirus 2 (SARS-CoV-2), responsible for the COVID-19 pandemic and with the potential to affect several mammal species, there is an increased concern about the risk of infection in aquatic mammals. The goal of this study was to molecularly screen the presence of morbillivirus and coronavirus infections in cetaceans stranded in several regions of the Brazilian coast in order to determine their occurrence rates, pathogenicity, and range of potentially susceptible cetacean species. We molecularly tested tissue samples of 118 cetaceans, belonging to 20 species, found stranded in Brazil, between 2015 and 2022. Overall, 2.5% (3/118) of the analyzed cetaceans were positive for GDMV infection: a Guiana dolphin (*Sotalia guianensis*), an Atlantic spotted dolphin (*Stenella frontalis*), and a humpback whale (*Megaptera novaeangliae*). None of the animals were positive for coronavirus. Our findings indicate that the morbillivirus sequence type identified in Indo-Pacific bottlenose dolphins (*Tursiops aduncus*) of Australia and our GDMV sequences from Brazil belong to the same strain. The systematic monitoring of cetacean morbilliviruses is recommended to properly estimate the occurrence rate, pathogenicity and evolution of these viruses, which may help anticipate novel epizooties and reduce their impact on endangered cetacean populations.

## Introduction

The Brazilian coast is one of the largest in the world, sustaining a rich biodiversity, with descriptions of over 45 cetacean species; nine mysticetes (i.e., baleen whales) and 36 odontocetes (e.g., dolphins and porpoises) [1]. Due to their ecologic and physiologic adaptations to a fully aquatic life, cetaceans are considered environmental sentinels of the marine environment [2]. Therefore, the study of emerging and reemerging infectious diseases in these animals is crucial to evaluate the health of their ecosystems, subjected to several threats over the last decades [3,4].

*Morbillivirus ceti* (syn. *Cetacean morbillivirus* [CeMV], genus *Morbillivirus*, family *Paramyxoviridae*) is an enveloped negative-sense single-stranded RNA virus considered an important cause of morbidity and mortality in cetaceans and pinnipeds worldwide [5]. Seven strains of CeMV are recognized to date: Dolphin morbillivirus (DMV) [6], Porpoise morbillivirus (PMV) [7], Pilot whale morbillivirus (PWMV) [8,9], Beaked whale morbillivirus (BWMV) [10], Guiana dolphin morbillivirus (GDMV) [11], one strain found in Indo-Pacific bottlenose dolphins (*Tursiops aduncus*) [12], and the Fraser dolphin morbillivirus [13]. In Brazil, two morbillivirus strains have been reported in cetaceans: GDMV and PWMV [14–20]. GDMV was detected in multiple cetacean species [14–18], including in an unusual mortality event (UME) that killed at least 270 Guiana dolphins (*Sotalia guianensis*) in Rio de Janeiro state, between November 2017 and March 2018 [19]. PWMV infections were reported in short-finned pilot whales (*Globicephala macrorhynchus*) of Brazil in 2020 [20], indicating that other strains also circulate in Brazilian waters. Despite these reports, the current knowledge regarding morbillivirus infection occurrence rate, pathogenesis, and epidemiology in Brazil is still scarce.

Coronaviruses, subfamily *Letovirinae*, family *Coronaviridae*, order *Orthocoronavirus*, are enveloped positive-sense single-stranded RNA viruses divided into four genera: *Alpha*-, *Beta*-, *Gamma*-, and *Deltacoronavirus*. Coronavirus infection can affect several systems (e.g., respiratory, gastrointestinal, nervous) in a broad range of mammal and bird species [21–23]. Coronaviruses are considered important emerging infectious agents due to their potential to switch hosts and also due to their zoonotic potential, as seen in the severe acute respiratory syndrome (SARS) and the COVID-19 pandemic [22,24]. In cetaceans, there is limited information regarding coronavirus infection, with few reports of gammacoronavirus infection in captive and free-ranging odontocetes [25–27]. Clinical signs and histopathologic examination of cetaceans infected with gammacoronaviruses portrayed gastrointestinal problems (e.g., diarrhea) in Atlantic bottlenose dolphins (*Tursiops truncatus*) [26], and pulmonary lesions, and hepatic failure due to hepatic necrosis in beluga whales (*Delphinapterus leucas*) [25].

A broad investigation of morbillivirus and coronaviruses infection in wild marine mammals is essential to shed light into the pathogenicity and epidemiological aspects of these infections, and to understand these viruses dynamics and potential impacts in cetacean populations [5,28]. Additionally, following the 2017–2018 GDMV-associated UME reported in Brazil [19], there is an increasing concern about the impact of infectious diseases on cetacean conservation, particularly on threatened species. Therefore, applying a One Health approach to the study of infectious agents in marine mammals of Brazil is an essential conservation measure. The objective of this study was to survey morbillivirus and coronavirus in a large panel of cetacean species occurring in different regions of Brazil. We also evaluated other important epidemiological aspects: the occurrence rate of these infections, viral detection in novel susceptible species, the circulating viral strains, and their pathogenicity.

## Materials and methods

### Samples

The selection criteria used herein aimed on a broad diversity of cetacean species and stranding locations. Thus, we selected individuals of resident species (Guiana dolphin, Lahille’s dolphin [*Tursiops truncatus gephyreus*] and Franciscana [*Pontoporia blainvillei*]) from different coastal regions, including animals with signs of infectious processes, animals that died due to anthropogenic interactions, and also individuals of non-resident species that occur less frequently in the studied region.

Overall, we selected 118 cetaceans, comprising 20 species, that stranded between November 2015 and January 2022, in the northeastern (25/118), southeastern (64/118) and southern (29/118) regions of Brazil. These individuals were either found dead or stranded alive and died under treatment; or died shortly after admission into rehabilitation centers. Animals were selected according to their decomposition condition status (DCS) (from fresh to moderate autolysis) [29] and unusual records of the species. Cases in advanced autolysis were included if they were found in an underrepresented region or belonged to an undersampled species (*e*.*g*., humpback whale *Megaptera novaeangliae*, in the Abrolhos Archipelago, northeastern Brazil). All individuals that were mummified or only presented the skeleton were excluded. Age class (*i*.*e*., fetus, calf, juvenile and adult) was stablished based on total body length [30], and characteristics such as the presence of vibrissae, and sexual maturity were confirmed by histopathology analyzes. All 118 animals were tested for paramyxovirus infection (618 analyzed tissue samples), while 93 cetaceans were tested for coronavirus (345 analyzed tissue samples). The epidemiological and biological data of the tested individuals are summarized in S1 Table.

### Pathological analyses

Samples for histological (10% neutral buffered formalin) and molecular analyses (frozen at -20 °C and stored at -80 °C until processing) were collected during standardized necropsies (29). RT-PCR-positive cases were also evaluated by histology (hematoxylin and eosin).

### Molecular analyzes

The following frozen samples from each animal were selected for the molecular screening of coronavirus and morbillivirus: cerebrum, cerebellum, brainstem, spinal cord, kidney, liver, lung, mesenteric lymph node, prescapular lymph node, pulmonary lymph node, small intestine, spleen, feces. Total RNA extraction was performed with TRIzol-LS (Life Technologies Corporation, CA, USA), and reverse transcription reaction was performed using random primers and M-MLV Reverse Transcriptase (Life Technologies Corporation).

For paramyxovirus and coronavirus screening, two consensus nested broad-range RT-PCRs were performed to amplify a 530 base pair (bp) fragment of the RNA dependent RNA polymerase (RdRp) gene of *Paramyxoviridae*—including *Morbillivirus* and other paramyxovirus genera (RdRp-PAR-nPCR) [31], and a 432 bp fragment of coronavirus *RdRp* (RdRp-COV-nPCR [32]. Paramyxovirus-positive samples were further tested using a RT-PCR directed to the phosphoprotein (P) gene of the genus *Morbillivirus* (P-MV-nPCR), yielding a 420 bp fragment, in order to confirm morbillivirus infection and strain typing [7]. In RT-PCR-positive animals, all the available tissues were tested by RdRp-PAR-nPCR and RdRp-COV-nPCR, and subsequently tested by P-MV-nPCR, if applicable.

Amplicons of the expected size obtained with the P-MV-nPCR were purified using PureLink^™^ Quick Gel Extraction Kit (Life Technologies Corporation). Both strands were directly sequenced by Sanger using the ABI PRISM BigDye^®^ Terminator v3.1 kit (Ready Reaction Cycle Sequencing, Applied Biosystems, Foster City, USA), and assembled using the Codon Code aligner v.4.2.1 software (Codon Code Corp. Dedham, USA). The obtained consensus sequences of partial P genes were queried for similarity using the Basic Local Alignment Search Tool (BLAST). Subsequently, the alignments with other CeMV P gene sequences available were conducted using Mega 7 [33]. The deduced CeMV amino acid P sequences obtained in this study, along with the selected CeMV P sequences in GenBank/EMBL/DDBJ database were used for inferring molecular phylogeny, totalizing 32 sequences. Phocine distemper virus was selected as outgroup. Phylogenetic analyzes were conducted in MEGA7 software. In addition, a fragment of 164 nucleotides of the P gene of 107 CeMV sequences was used to evaluate the PMV, DMV, BWMV, GDMV and PWMV intra and inter strain diversity, based on pairwise identity distance (Table 1). That 164 bp fragment of the P gene was chosen to increase the representation of a large number of CeMV strains, once it is available for most of the sequences in GenBank. For the GDMV strain, the intra-group variability was also verified using a longer fragment (206–389 nucleotides in length and 70–130 amino acids in length), available for this variant in GenBank. The nucleotide and amino acid substitutions are shown in Table 1. The alignments, evolutionary and diversity analyzes were conducted in MEGA7 software. The sequences obtained in this study were compared with those found in Indo-Pacific bottlenose dolphins of western Australia, based on p-distance.

**Table 1:** Substitutions observed in P gene nucleotide (nt) and amino acid (aa) sequences among all the GDMV reported in Brazil and the CeMV detected in Swan River, Australia, in comparison with strain MQ904P (MG845552), which was detected during an unusual mortality event in a Guiana dolphin (*Sotalia guianensis*) population, Brazil.

Animal ID	Stranding date/ sampling collection	Cetacean species	GenBank accession number	Nt phosphoprotein gene sequence size (bp)	Position of nt substitutions related to MG845552	Aa phosphoprotein gene sequence size	Position of aa substitutions related to MG845552	Reference
Swan River	2009	*Tursiops aduncus*	N/D	389	374 (A/G)	130	126 (E/G)	Jacob et al. (10); Stephens et al. (12)
Strain Ea2010	2010, Sep 14	*Eubalaena australis*	MH497060	213	1 (T/G)	72	1 (C/G)	Groch et al. (15)
Strain Sg-2010	2010, Nov 30	*Sotalia guianensis*	KF711855	374	374 (A/G)	125	-	Groch et al. (11)
Strain Mn-B10	2011, Sep 22	*Megaptera novaeangliae*	MT799694	212	-	72	-	Groch et al. (17)
Strain Mn-B26	2012, Aug 28	*Megaptera novaeangliae*	MT799695	212	-	72	-	Groch et al. (17)
Strain MM710_Kidney	2014, Aug	*Orcinus orca*	MT647723	206	12 (G/A), 18(G/A)	70	6 (G/R), 8 (A/T)	Groch et al. (14)
Strain Ea2015	2015, Aug 1	*Eubalaena australis*	MH497061	213	1 (T/G)	72	1 (C/G)	Groch et al. (15)
Strain MQ904P (UME)	2017, Nov	*Sotalia guianensis*	MG845551	405	150 (C/T)	135	51 (S/P)	Groch et al. (18)
Strain 318_19 (case 1)	2018, Apr 24	*Stenella frontalis*	This study	377	-	126	-	This study
Strain 315_19_ (case 2)	2018, Nov 22	*Sotalia guianensis*	This study	377	-	126	-	This study
Strain 159_21 (case 3)	2020, Dec 10	*Megaptera novaeangliae*	This study	377	97 (A/G)	126	34 (K/E)	This study

N/D: Sequence not deposited at the GenBank database, but published by Jacob et al. [10].

### Permits

The field studies and sample collections were performed in full compliance with specific federal permits issued by the Brazil Ministry of Environment (MMA) and the Chico Mendes Institute for Biodiversity Conservation (ICMBio), under the Biodiversity Information and Authorization System (SISBIO 69115–4) and National System of Genetic Resource Management and Associated Traditional Knowledge (SISGEN ADA22DD), all in accordance with the Ethic Committee on Animal Use of the School of Veterinary Medicine and Animal Sciences (University of São Paulo)–CEUA/FMVZ (certificate number 6819150419). Consent to participate: not applicable.

## Results

### Samples

Herein, we analyzed 57 males, 58 females, and three cetaceans of undetermined sex (due to their advanced decomposition status, *e*.*g*., predation), identified as adults (n = 58), juveniles (n = 41), calves (n = 17), and two fetuses (S1 Table).

### Molecular findings

**Fig 1:**
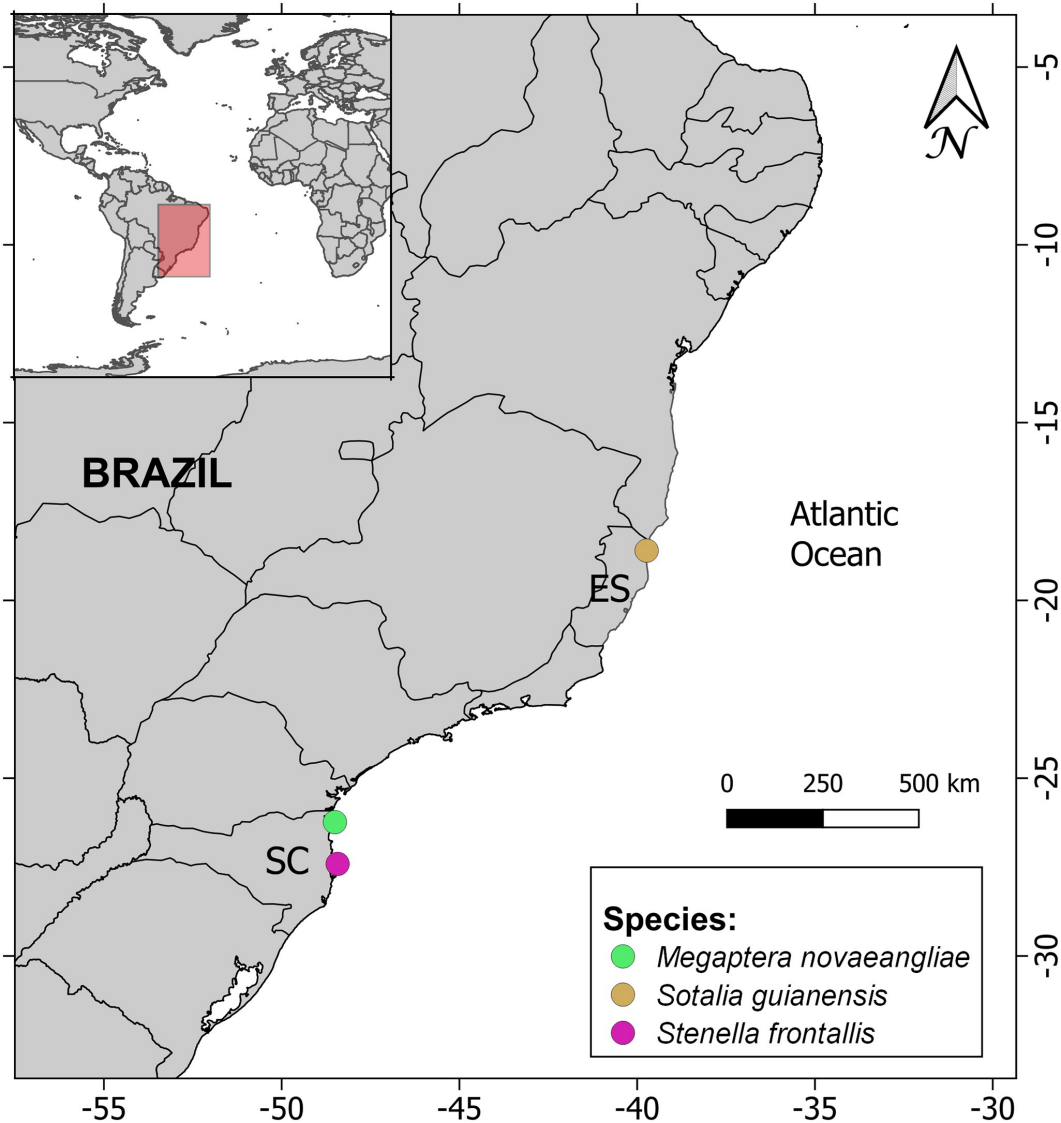
Geographical locations of the individuals that were RT-PCR-positive for Guiana dolphin morbillivirus (GDMV) along the Brazilian coast. ES = Espírito Santo state, SC = Santa Catarina state.

Three out of 118 individuals (2.5%) were positive for CeMV: (i) an Atlantic spotted dolphin (*Stenella frontalis*, case 1) stranded in Florianópolis, Santa Carina state (southern region), which tested positive in brain, tongue and kidney; (ii) a Guiana dolphin (case 2) stranded in São Mateus, Espírito Santo state (southeastern region), positive in prescapular lymph node; and (iii) a humpback whale (case 3) stranded in São Francisco do Sul, Santa Catarina state (southern region), which tested positive in brain and lungs (Fig 1). According to cetacean species, one out of five Atlantic spotted dolphins (1/5), 3.6% in Guiana dolphins (1/28) and 6.3% in humpback whales (1/16) were morbillivirus-RT-PCR-positive. None of the individuals tested positive to coronavirus.

The retrieved P gene nucleotide sequences (377 bp length) of the three cases were identical amongst them, except for a synonymous substitution (A/G) in position 97 of the sequenced fragment obtained from the humpback whale (case 3, Table 1). All samples were assigned as GDMV strain. The sequences obtained herein are highly similar to those from previous GDMV cases reported in Brazil, including those from the 2017–2018 UME (GenBank accession no. MG845551 and MG845552, Table 1). The substitutions observed in nucleotide and amino acid sequences of the P gene among all the GDMV reported in Brazil in relation to the sequence type MQ904P (MG845551) are shown in Table 1. The GDMV P gene sequences obtained in this study in the humpback whale, the Guiana dolphin and the Atlantic spotted dolphin were submitted to GenBank/DDBJ/ENA database under accession numbers PP475487, PP475488, and PP475489, respectively.

**Fig 2:**
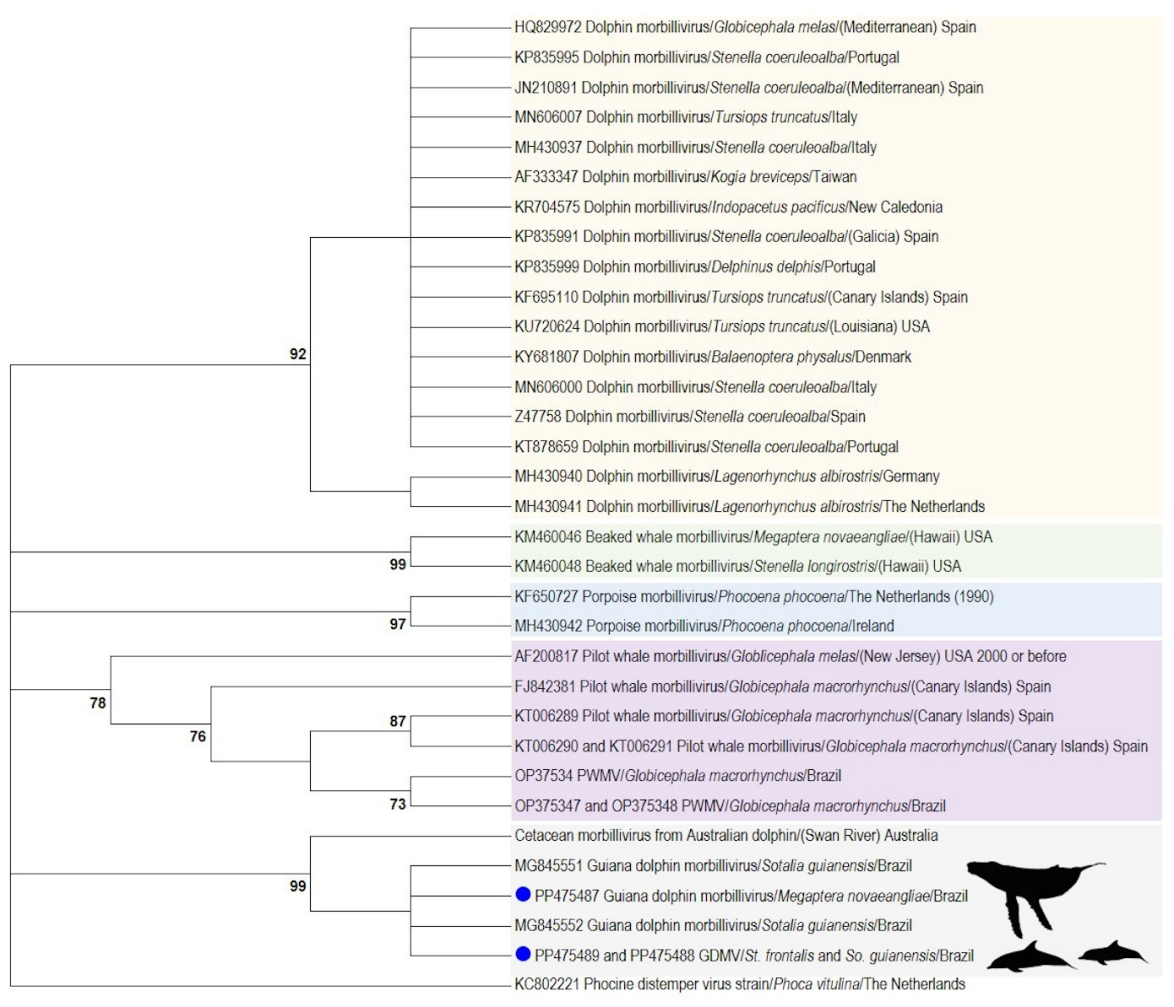
Maximum-likelihood phylogenetic tree based on Jones-Taylor-Thornton model with a discrete Gamma distribution of the alignment of the partial phosphoprotein gene amino acid sequences of morbillivirus (1) obtained in this study (blue dots), (2) other Guiana dolphin morbillivirus sequences (gray square), and (3) cetacean morbillivirus sequences representing the different lineages aside from Guiana dolphin morbillivirus recognized to this date—Dolphin morbillivirus (yellow square), Beaked whale morbillivirus (green square), Porpoise morbillivirus (blue square), Pilot whale morbillivirus (violate square)—Available at the GenBank/DDBJ/EMBL database. A phocine distemper virus phosphoprotein sequence was selected as outgroup. The bootstrap consensus tree inferred from 1000 replicates. Bootstrap values lower than 70 were omitted. GDMV: Guiana dolphin morbillivirus.

The phylogenetic tree based on the 118 amino acid fragment of the P gene of 32 cetacean morbillivirus sequences clearly classified our sequences within the Guiana dolphin morbillivirus strain, and also grouped them (99% bootstrap value) with a sequence retrieved from an Indo-Pacific bottlenose dolphin of the Swan river, Australia. The other cetacean morbillivirus sequences analyzed in the phylogram were also accurately classified (Fig 2).

The divergence analysis of the gene fragment (pairwise identity distance analysis) showed significant divergence between each group of viral strains (Table 2), corroborating with the phylogram. Nevertheless, low divergence was observed in the intra-group of CeMV variants using this molecular marker (Table 2). Considering the detected GDMV sequence types, the variability observed using the 181 bp fragment of the P gene was lower than the one observed when a longer fragment was analyzed (206–377 bp; Table 2). Based on P-distance, the morbillivirus P sequence of Indo-Pacific bottlenose dolphins of western Australia and the GDMV sequences of the same length (all three obtained in this study and two from Guiana dolphins MG845551 and MG845552) present high similarity (nucleotide identities ranging from 99.4 to 99.7% and amino acid similarities from 98.3% to 99.2%).

**Table 2:** Estimation of average evolutionary divergence of the phosphoprotein gene within and between each group of cetacean morbillivirus strains.

P-distance values (standard deviation)	GDMV	DMV	BWMV	PMV	PWMV
GDMV	0.0000 (0.0000)				
DMV	0.2532 (0.0352)	0.0057 (0.0018)			
BWMV	0.3023 (0.0359)	0.1717 (0.0294)	0.0016 (0.0012)		
PMV	0.2264 (0.0334)	0.1340 (0.0272)	0.1509 (0.0287)	0.0065 (0.0032)	
PWMV	0.2615 (0.0353)	0.1547 (0.0283)	0.2071 (0.0317)	0.1527 (0.0291)	0.0088 (0.0037)

GDMV: Guiana dolphin morbillivirus; DMV: Dolphin morbillivirus; BWMV: Beaked-whale morbillivirus; PMV: Porpoise morbillivirus; PWMV: Pilot whale morbillivirus.

### Pathological findings in GDMV-positive animals

**Fig 3:**
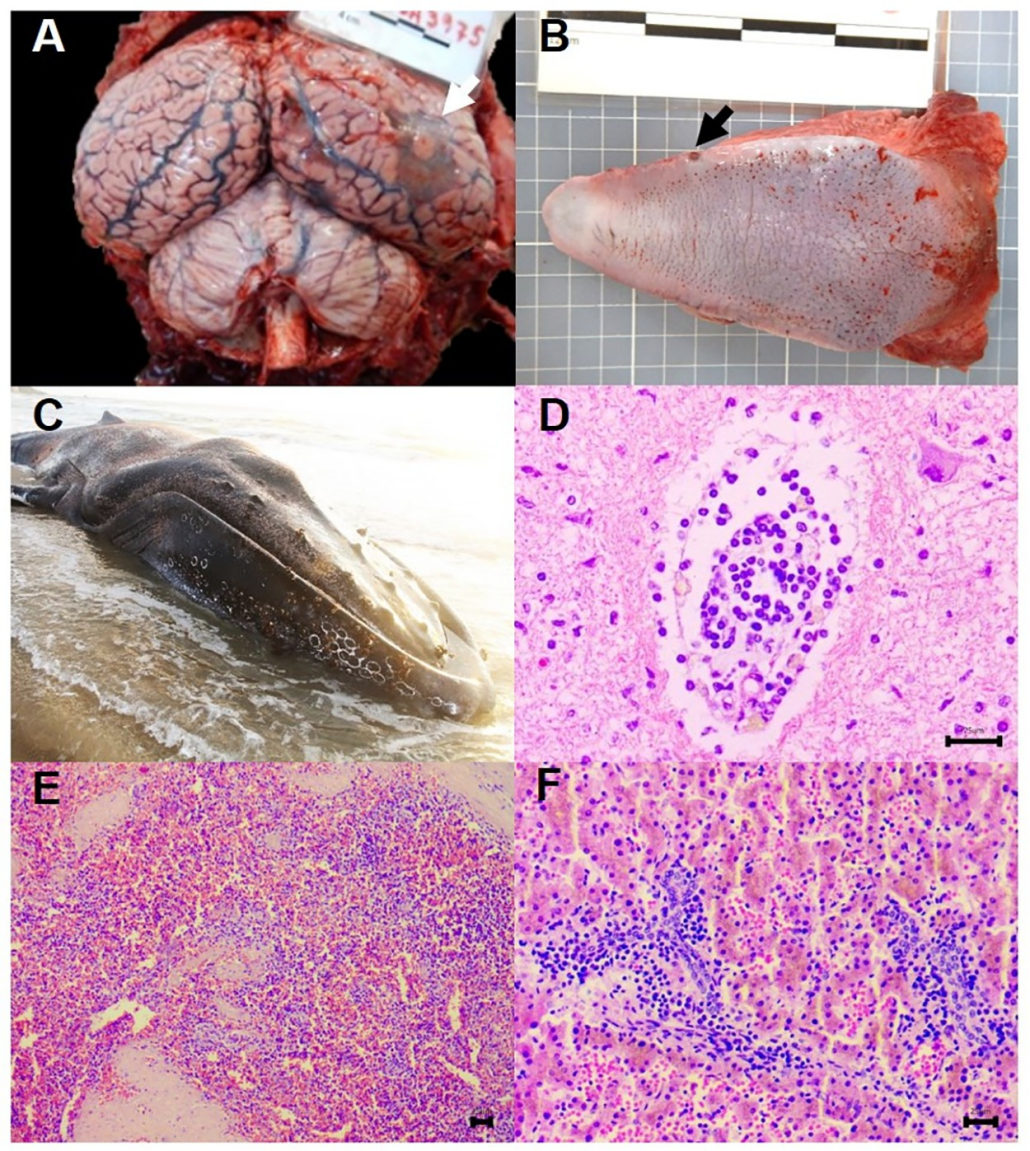
Pathological findings in GDMV-RT-PCR-positive cases. (A) Atlantic spotted dolphin (*Stenella frontalis*). Brain. Congestion, focal lesion in occipital cortex surrounded by an area of malacia (white arrow); (B) Atlantic spotted dolphin. Mild focal ulcerative lesion in the right edge of the tongue (arrow); (C) Humpback whale (*Megaptera novaeangliae*) stranded in poor body condition with moderate diffuse whale lice; (D) Humpback whale. Cerebrum presenting perivascular cuffs and mononuclear infiltrate; (E) Humpback whale. Note the lymphoid depletion and the disorganization of the splenic parenchyma. (F) Humpback whale. Observe the periportal lymphoplasmacytic-histiocytic hepatitis, mild hepatic congestion and mild amount of brown intracytoplasmic pigment.

Case 1, the Atlantic spotted dolphin, presented good body condition. The main gross findings were generalized congestion, moderate diffuse pulmonary distention and congestion, presence of mild to moderate multifocal necrosis areas in occipital cortex (Table 3, Fig 3A), and a mild focal ulcerative lesion in the dorsal aspect of the tongue (Fig 3B). Additionally, *postmortem* linear cuts were observed in the caudal fin and abdominal region, with organ exposure, suggesting anthropic interaction. Case 2, the Guiana dolphin, was in advanced autolysis, which hampered its examination. Finally, Case 3, the humpback whale, stranded alive at the end of the reproductive season (in Brazil, from July to November), and died soon after. Upon external examination, the animal was in poor body condition, with diffuse moderate whale lice (*Cyamus boopis*) infestation (Fig 3C), mild presence of barnacles, and mild multifocal coockiecutter shark (*Isistius* sp.) bites in different scar stages. This individual also presented moderate diffuse petechiae and congestion of cerebrum and cerebellum, with perivascular cuffs of mononuclear infiltrate (Fig 3D), diffuse pleural thickness and pulmonary congestion, moderate diffuse hepatic congestion, and a focal nodule in the mucosal layer of the bladder. The histopathological findings are described in Table 3.

**Table 3:** Epidemiological and pathological data of the morbillivirus-RT-PCR-positive cetaceans detected in this study.

Case nº	Species	Stranding date	Location	Sex	Age class	TBL (m)*	Decomposing status	Histological analysis
1	*Stenella frontalis*	Apr 24, 2018	Florianópolis, SC	Male	Adult	1.83	Fresh	**Lungs:** moderate multifocal to coalescent suppurative bronchopneumonia. **Lymph node:** moderate diffuse granulocytic lymphadenitis. Mild to moderate diffuse lymphoid hyperplasia. **Brain:** moderate to marked focally extensive encephalomalacia with hemorrhagic areas and phagocytizing glial cells associated with the presence of parasite eggs compatible with *Nasitrema* sp. **Heart, skeletal muscle, liver, diaphragm, kidney, adrenal gland:** NSFO** and partial autolysis.
2	*Sotalia guianensis*	Nov 22, 2018	São Mateus, ES	Female	Adult	1.63	Autolysis	Advanced autolysis
3	*Megaptera novaeangliae*	Dec 10, 2020	São Francisco do Sul, SC	Male	Juvenile	12.20	Fresh	**Adrenal gland:** moderate multifocal lymphocytic adrenalitis. **Brain:** mild multifocal lymphocytic meningoencephalitis with perivascular cuffs, mild multifocal lymphocytic myelitis with perivascular cuffs. **Stomach:** moderate multifocal lymphocytic gastritis. **Liver:** moderate multifocal periportal lymphoplasmacytic-histiocytic hepatitis, mild congestion., **Intestine:** enteritis with mild hyperplasia of globet cells and severe villous atrophy. **Lymph node:** moderate lymphoid depletion in lymph node and spleen. **Colon**: mild multifocal neutrophilic colitis. **Humerus**: focal necrosis in humeral head.

*TBL: Total body length.

**NSFO: No significant findings were observed.

## Discussion

The evaluation of a large diversity of cetacean species conducted in the present study allowed the identification of the first GDMV infection potentially associated with tissue lesions in humpback whales (case 3), and the first morbillivirus infection in Atlantic spotted dolphins (case 1) presenting compatible pathological findings, expanding the host range of species susceptible to GDMV. Previous GDMV infections were reported in exhaled breath of humpback whales and in tissue samples of Guiana dolphins, a killer whale (*Orcinus orca*), and southern right whales (*Eubalaena australis*) [14–18]. Further serological surveys are required in order to understand the exposure of cetaceans against GDMV in Brazil.

The GDMV detection rate in the present study was 2.5% (3/118). Studies conducted worldwide reported variable values of CeMV infection rates (ranging from 1.8% to 31.9%) in areas that suffered UMEs [34–36]; however, such discrepancies between occurrence rates are expected. Both GDMV and DMV strains are able to infect several cetacean species, but circulate in different regions (e.g. GDMV in Southeastern Atlantic and likely in the Indian Ocean, based on the analysis of the sequence from Australia, and DMV in the Northern Atlantic and Mediterranean Sea) and may have distinct pathogenic features and host susceptibility. Characteristics of the cetacean populations used in each study may also contribute to these discrepancies, such as the diversity of cetacean species sampled (some species seem to be highly susceptible to CeMV infections, such as striped dolphins), and the eligibility criteria used for sampling (*e*.*g*., carcass decomposition stage, animals with/without viral related lesions). In addition, the distinct diagnostic methods and protocols used in these studies should also be considered. In Brazil, a high morbillivirus occurrence rate (27.5%, 40/325) was reported in Guiana dolphins of Paraná state, southern Brazil, between February 2016 and November 2018 [16]. Nevertheless, the diagnosis was based solely on tissue antigen detection through immunohistochemistry, and none of the positive cases were confirmed by RT-PCR, which limits direct comparisons with the results obtained herein. The GDMV-positive animals detected in this study were collected in northeastern, southeastern and southern Brazil, indicating this virus’ circulation along the Brazilian coast [11,14,15,17].

The Atlantic spotted dolphin (case 1) had an ulcerative lingual lesion similar to those described in morbillivirus-infected cetaceans [37]. Additionally, this individual had an extensive area of parasitic malacia in the central nervous system, which along with the morbillivirus infection, may have contributed to its stranding. The impact of this strain over the Atlantic spotted dolphin population is still unknown. Of note, the Atlantic spotted dolphin population of the Santos Basin is one of the largest cetacean populations in the area, with 26,909 estimated individuals (personal communication with the Cetacean Monitoring Program-Santos Basin, Brazil).

BWMV was the first detected CeMV strain in humpback whales, reported in a stranded animal in the USA, in 1998 [10]. Another CeMV strain—GDMV, was detected in the exhaled breath of apparently healthy adult humpback whales (2/48 groups of whales), sampled in 2011 and 2012, in a study conducted in the Abrolhos Bank, northeastern Brazil [17], and recently, in tissue samples of two juvenile males of that species stranded in southern Brazil, in 2022 [18]. Herein, the GDMV-positive humpback whale (case 3) presented lesions suggestive of morbillivirus infection, such as bronchopneumonia and non-suppurative encephalitis, consistent with an acute GDMV infection. The animal stranded in poor body condition and presented high whale lice infestation, which indicates impaired locomotion. Our whale stranded in 2020, two years after the Rio de Janeiro UME [19], and almost 10 years after the GDMV detection in exhaled humpback whale breath [17]. Thus, our findings, alongside those reported by de Amorim et al. (2024), suggest that GDMV circulates in the humpback whale population of Brazil (Breeding stock A), which sustains high site fidelity to breeding areas of the southwestern Atlantic.

GDMV infection with associated lesions was reported in Guiana dolphins in two previous studies in Brazil [11,19]. Herein, we tested 28 Guiana dolphins from Ceará, Espírito Santo, São Paulo, and Santa Catarina states, and found one positive individual (case 2) that stranded in November 2018, in São Mateus, Espírito Santo (northeastern region). Of note, this animal was found dead in the same location as the first GDMV case reported in Brazil [11]—a Guiana dolphin that stranded in 2010, suggesting that GDMV is recurrently circulating in Guiana dolphins. The studied animal died in the same year of the Rio de Janeiro UME, but over 350 km away.

The GDMV found in Brazil clustered with a high support with a morbillivirus detected in an Indo-Pacific bottlenose dolphin in western Australia, which was previously considered a different strain [12] and was not present in public databases, but published by Jacob et al. [10]. When compared with the closest GDMV phosphoprotein sequences of the same size, the sequence from Australia presented only a single amino acid substitution with the closest GDMV sequences, the same difference that we observed between different GDMV phosphoprotein sequences. Therefore, the GDMV and the Australian sequence are likely comprised within the same viral strain. Our findings suggest that GDMV circulates in the southern Atlantic and the Indian Ocean, although the analyzes of other genes is recommended to confirm this possibility.

Despite the reports of some systemic infections in the Mediterranean sea, most DMV infections outside outbreak periods were associated with the chronic encephalitic form, especially in striped dolphin populations [5,28,34,36,38–42]. There is no available information regarding the extent of the GDMV strain association with restricted central nervous system (CNS) infections. In our study, all the positive cases presented systemic infections; however, only 64 of the 118 studied individuals had samples of CNS available for molecular testing, which may have underestimated the number of obtained positives if they presented chronic encephalitic forms [43]. According to immunohistochemical analysis, the GDMV found in the Guiana dolphins of the Brazilian outbreak showed lower neurotropism and less severe pathological findings than those observed in dolphins with DMV infections [44]. The impact of chronic encephalic infections on virus transmission and endemicity has been discussed, but is still unknown [43].

Several large-scale outbreaks have been reported in the Mediterranean Sea, with two main types of DMV identified according to the geographical origin—the Mediterranean and northeastern (NE) Atlantic, with the latter gradually replacing the former, possibly due to the overlapping of migrating cetacean species coming from the Atlantic [36]. The introduction of the new NE-Atlantic DMV lineage in the Mediterranean Sea into an immune naïve cetacean population seems to explain, at least in part, the frequent epizooties in the region [34,36,42,45]. In Brazil, there is no high divergence among the detected GDMV sequence types (based only on partial P gene sequences), which should be one of the explanations for the infrequent outbreaks in the region. Nevertheless, one should consider that the low variability observed in Brazilian sequence types may also reflect the reduced number of samples available for analysis. It is a matter of debate if the GDMV strain is endemic in resident cetacean populations (*e*.*g*., Guiana dolphins) or if it has been sporadically introduced into Brazilian waters by other cetacean species that migrate to the region, especially because we have no serological data regarding anti-morbillivirus antibodies in cetaceans of this country. Nevertheless, the low number of outbreaks occurring in the region suggests some degree of immunity, characteristic of endemic infections. There are several locations considered relevant in terms of cetacean conservation in the Brazilian coast, including breeding grounds for humpback whales like the Abrolhos Bank [46]. Of note, other locations, particularly coastal waters, sustain important host resident cetacean populations: Lahille’s bottlenose dolphin (*Tursiops truncatus gephyreus*) [47], Guiana dolphin [48], and franciscana—the latter classified as the most endangered cetacean in the southwestern Atlantic Ocean [49]. This study evaluated 25 franciscanas, 28 Guiana dolphins and one Lahille’s bottlenose dolphin; however, only one Guiana dolphin was GMDV-positive. To this date, there are no reports of morbillivirus infections in franciscana or in Lahille’s bottlenose dolphin.

Although some studies suggested high susceptibility of certain cetacean species to SARS-CoV-2 based on the sequence similarity between theirs and humans’ ACE-2 receptor [50,51], neither SARS-CoV-2 nor any other coronavirus were detected in this study. The three reports of gammacoronaviruses in cetaceans were from captive and free-ranging animals [25–27]. We should also emphasize that in a previous report, coronavirus viral nucleic acids were detected in feces, liver and heart samples [26–28], and herein, mesenteric lymph node (n = 39)/intestinal content (n = 4) were available for analysis in a reduced number of animals, which could explain the observed negative results.

Our findings emphasize the importance of implementing long-term systematic CeMV surveillance in Brazil to determine the occurrence rate of infection and to monitor viral evolution, in order to anticipate epizootic events and their impacts on the conservation of threatened cetacean populations. Further CeMV whole genome sequencing studies are required, particularly regarding poorly characterized strains as GDMV. Regarding coronaviruses, despite the negative results, the evaluation of fecal samples, especially of animals with diarrhea or undergoing rehabilitation should be considered in beach monitoring approaches conducted in Brazil.

## Supporting information

S1 Table Epidemiological and biological data (common name, species, age class, sex and region of stranding) of the cetaceans stranded on the Brazilian coast, and tested for morbillivirus and coronavirus. (XLSX)

## References

[1] Lodi L, Borobia M. In: Lodi L. e Borobia M, editors. Baleias, Botos e Golfinhos do Brasil. Rio de Janeiro: Technical Books Editora; 2013. pp. 478.

[2] Bossart GD. Marine mammals as sentinel species for oceans and human health. Vet Pathol. 2011;48(3):676–90. doi: 10.1177/0300985810388525 21160025

[3] Van Bressem M-F, Raga JA, Di Guardo G, Jepson PD, Duignan PJ, Siebert U, et al. Emerging infectious diseases in cetaceans worldwide and the possible role of environmental stressors. Dis Aquat Organ. 2009 Sep 23;86(2):143–57. doi: 10.3354/dao02101 19902843

[4] Sacristán C, Ewbank AC, Duarte-Benvenuto A, Sacristán I, Zamana-Ramblas R, Costa-Silva S, et al. Survey of selected viral agents (herpesvirus, adenovirus and hepatitis E virus) in liver and lung samples of cetaceans, Brazil. Sci Rep. 2024 Feb 1;14(1):2689. doi: 10.1038/s41598-023-45315-9 38302481 PMC10834590

[5] Duignan PJ, Van bressem MF, Cortés-Hinojosa G, Kennedy-Stoskopf S. Viruses. In: Gulland F, Dierauf L, Whitman K, editors. CRC handbook of marine mammal medicine. 3rd edition. Boca Raton: CRC Press; 2018. pp. 331–56.

[6] Domingo M, Ferrer L, Pumarola M, Marco A, Plana J, Kennedy S, et al. Morbillivirus in dolphins. Nature. 1990 Nov;348(6296):21–21. doi: 10.1038/348021a0 2234055

[7] Barrett T, Visser IK, Mamaev L, Goatley L, van Bressem MF, Osterhaust AD. Dolphin and porpoise morbilliviruses are genetically distinct from phocine distemper virus. Virology. 1993 Apr;193(2):1010–2. doi: 10.1006/viro.1993.1217 8460473

[8] Taubenberger JK, Tsai MM, Atkin TJ, Fanning TG, Krafft AE, Moeller RB, et al. Molecular Genetic Evidence of a Novel Morbillivirus in a Long-Finned Pilot Whale (*Globicephalus melas*). Emerg Infect Dis. 2000 Feb;6(1):42–5. 10653568 10.3201/eid0601.000107 PMC2627976

[9] Bellière EN, Esperón F, Fernández A, Arbelo M, Muñoz MJ, Sánchez-Vizcaíno JM. Phylogenetic analysis of a new Cetacean morbillivirus from a short-finned pilot whale stranded in the Canary Islands. Res Vet Sc. 2011 Apr;90(2):324–8. doi: 10.1016/j.rvsc.2010.05.038 20576281

[10] Jacob J, West K, Levine G, Sanchez S, Jensen B. Initial characterization of novel beaked whale morbillivirus in Hawaiian cetaceans. Dis Aquat Organ. 2016 Jan 13;117(3):215–27. doi: 10.3354/dao02941 26758655

[11] Groch KR, Colosio AC, Marcondes MCC, Zucca D, Díaz-Delgado J, Niemeyer C, et al. Novel Cetacean Morbillivirus in Guiana Dolphin, Brazil. Emerg Infect Dis. 2014 Mar;20(3):511–3. doi: 10.3201/eid2003.131557 24565559 PMC3944878

[12] Stephens N, Duignan PJ, Wang J, Bingham J, Finn H, Bejder L, et al. Cetacean Morbillivirus in Coastal Indo-Pacific Bottlenose Dolphins, Western Australia. Emerg Infect Dis. 2014 Apr;20(4):672–6. doi: 10.3201/eid2004.131714 24656203 PMC3966363

[13] West KL, Silva-Krott I, Landrau-Giovannetti N, Rotstein D, Saliki J, Raverty S, et al. Novel cetacean morbillivirus in a rare Fraser’s dolphin (*Lagenodelphis hosei*) stranding from Maui, Hawai‘i. Sci Rep. 2021 Dec 9;11(1):15986. 34373473 10.1038/s41598-021-94460-6 PMC8352961

[14] Groch KR, Jerdy H, Marcondes MC, Barbosa LA, Ramos HG, Pavanelli L, et al. Cetacean Morbillivirus Infection in a Killer Whale (*Orcinus orca*) from Brazil. J Comp Pathol. 2020;181. 10.1016/j.jcpa.2020.09.012 33288147

[15] Groch KR, Groch KR, Kolesnikovas CKM, de Castilho P V., Moreira LMP, Barros CRMB, et al. Cetacean morbillivirus in Southern Right Whales, Brazil. Transbound Emerg Dis. 2019 Jan 15;66(1):606–10. doi: 10.1111/tbed.13048 30365233

[16] Cunha HA, Santos-Neto EB, Carvalho RR, Ikeda JMP, Groch KR, Díaz-Delgado J, et al. Epidemiological features of the first Unusual Mortality Event linked to cetacean morbillivirus in the South Atlantic (Brazil, 2017–2018). Mar Mammal Sci. 2021 Oct 22;37(4):1375–90.

[17] Groch KR, Blazquez DNH, Marcondes MCC, Santos J, Colosio A, Díaz Delgado J, et al. Cetacean morbillivirus in Humpback whales’ exhaled breath. Transbound Emerg Dis. 2021 Jul 30;68(4):1736–43. doi: 10.1111/tbed.13883 33070446

[18] de Amorim DB, de Camargo LJ, Ribeiro PR, Budaszewski R da F, Menegatt JCO, Paz MC, et al. Characterization of Cetacean Morbillivirus in Humpback Whales, Brazil. Emerg Infect Dis. 2024 Jun;30(6):1296–8. doi: 10.3201/eid3006.231769 38781986 PMC11138977

[19] Groch KR, Santos-Neto EB, Díaz-Delgado J, Ikeda JMP, Carvalho RR, Oliveira RB, et al. Guiana Dolphin Unusual Mortality Event and Link to Cetacean Morbillivirus, Brazil. Emerg Infect Dis. 2018 Jul;24(7):1349–54. doi: 10.3201/eid2407.180139 29912687 PMC6038766

[20] Costa-Silva S, Sacristán C, Soares RM, Carvalho VL, Castilho P V, Cremer MJ, et al. Short-Finned Pilot Whale Strandings Associated with Pilot Whale Morbillivirus, Brazil. Emerg Infect Dis. 2023 Jan;29(1):214–7. doi: 10.3201/eid2901.221549 36573734 PMC9796215

[21] Balasuriya UBR. Coronaviridae. In: McVey S, Kennedy M, Chengappa MM, editors. Microbiologia Veterinária. 3rd edition. Rio de Janeiro: Guanabara Koogan; 2017. pp. 465–83.

[22] Huang W-H, Teng L-C, Yeh T-K, Chen Y-J, Lo W-J, Wu M-J, et al. 2019 novel coronavirus disease (COVID-19) in Taiwan: Reports of two cases from Wuhan, China. J Microbiol Immunol Infect. 2020 Feb;53(3):481–34 doi: 10.1016/j.jmii.2020.02.009 32111449 PMC7102546

[23] do Vale B, Lopes AP, Fontes M da C, Silvestre M, Cardoso L, Coelho AC. Bats, pangolins, minks and other animals—villains or victims of SARS-CoV-2? Vet Res Commun. 2021 Feb 19;45(1):1–19. doi: 10.1007/s11259-021-09787-2 33464439 PMC7813668

[24] Zhu N, Zhang D, Wang W, Li X, Yang B, Song J, et al. A Novel Coronavirus from Patients with Pneumonia in China, 2019. N Engl J Med. 2020 Feb 20;382(8):727–33. doi: 10.1056/NEJMoa2001017 31978945 PMC7092803

[25] Mihindukulasuriya KA, Wu G, St. Leger J, Nordhausen RW, Wang D. Identification of a Novel Coronavirus from a Beluga Whale by Using a Panviral Microarray. J Virol. 2008 May 15;82(10):5084–8. doi: 10.1128/JVI.02722-07 18353961 PMC2346750

[26] Wang L, Maddox C, Terio K, Lanka S, Fredrickson R, Novick B, et al. Detection and Characterization of New Coronavirus in Bottlenose Dolphin, United States, 2019. Emerg Infect Dis. 2020;26(7):1610–2. doi: 10.3201/eid2607.200093 32568058 PMC7323548

[27] Legnardi M, Franzo G, Cecchinato M, Si H, Baston R, Mazzariol S, et al. First detection of gammacoronavirus in a striped dolphin (*Stenella coeruleoalba*) from the Adriatic Sea. Animals. 2024; 14(18): 2725. doi: 10.3390/ani14182725 39335313 PMC11429407

[28] Mira F, Rubio-Guerri C, Purpari G, Puleio R, Caracappa G, Gucciardi F, et al. Circulation of a novel strain of dolphin morbillivirus (DMV) in stranded cetaceans in the Mediterranean Sea. Sci Rep. 2019;9(1):1–9. 31278350 10.1038/s41598-019-46096-w PMC6611785

[29] Geraci JR, Lounsburry VJ. Marine mammal ashore. second. Baltimore: national aquarium in baltimore; 2005.

[30] Jefferson TA, Leatherwood S, Webber MA. In: Jefferson T. A.; Webber M. A.; Pitman RL, editor. Marine Mammals of the World: A comprehensive guide to their identification. 2nd edition. London: Academic Press/Elsevier; 2015. pp. 608.

[31] Tong S, Chern SW, Li Y, Pallansch MA, Anderson LJ. Sensitive and broadly reactive reverse transcription-PCR assays to detect novel paramyxoviruses. J Clin Microbiol. 2008. 46(8):2652–8. doi: 10.1128/JCM.00192-08 18579717 PMC2519498

[32] Holbrook MG, Anthony SJ, Navarrete-Macias I, Bestebroer T, Munster VJ, van Doremalen N. Updated and Validated Pan-Coronavirus PCR Assay to Detect All Coronavirus Genera. Viruses. 2021 Apr 1;13(4):599. doi: 10.3390/v13040599 33915875 PMC8067199

[33] Kumar S, Stecher G, Tamura K. MEGA7: Molecular Evolutionary Genetics Analysis Version 7.0 for Bigger Datasets. Mol Biol Evol. 2016. 33(7), 1870–1874. doi: 10.1093/molbev/msw054 27004904 PMC8210823

[34] Rubio-Guerri C, Jiménez MÁ, Melero M, Díaz-Delgado J, Sierra E, Arbelo M, et al. Genetic heterogeneity of dolphin morbilliviruses detected in the Spanish Mediterranean in inter-epizootic period. BMC Vet Res. 2018 Dec 24;14(1):248. doi: 10.1186/s12917-018-1559-0 30143035 PMC6109331

[35] Felipe-Jiménez I, Fernández A, Arbelo M, Segura-Göthlin S, Colom-Rivero A, Suárez-Santana CM, et al. Molecular Diagnosis of Cetacean Morbillivirus in Beaked Whales Stranded in the Canary Islands (1999–2017). Vet Sci. 2022 Mar 7;9(3):121. doi: 10.3390/vetsci9030121 35324849 PMC8950905

[36] Vargas-Castro I, Peletto S, Mattioda V, Goria M, Serracca L, Varello K, et al. Epidemiological and genetic analysis of Cetacean Morbillivirus circulating on the Italian coast between 2018 and 2021. Front Vet Sci. 2023 Jul 31;10(1). doi: 10.3389/fvets.2023.1216838 37583469 PMC10424449

[37] Duignan PJ, Geraci JR, Raga JA, Calzada N. Pathology of morbillivirus infection in striped dolphins (*Stenella coeruleoalba*) from Valencia and Murcia, Spain. 1992. Can J Vet Res. 1992. 56:242–248 1423061 PMC1263546

[38] Domingo M. Evidence for chronic morbillivirus infection in the Mediterranean striped dolphin (*Stenella coeruleoalba*). Vet Microbiol. 1995 May;44(2–4):229–39. 8588317 10.1016/0378-1135(95)00016-4

[39] Di Guardo G, Mazzariol S. Cetacean Morbillivirus-Associated Pathology: Knowns and Unknowns. Front Microbiol. 2016 Feb 8;7(FEB):1–5. doi: 10.3389/fmicb.2016.00112 26903991 PMC4744835

[40] Bento MCR de M, Eira CICS, Vingada JV, Marçalo AL, Ferreira MCT, Fernandez AL, et al. New insight into dolphin morbillivirus phylogeny and epidemiology in the northeast Atlantic: opportunistic study in cetaceans stranded along the Portuguese and Galician coasts. BMC Vet Res. 2016 Dec 26;12(1):176. doi: 10.1186/s12917-016-0795-4 27566667 PMC5002201

[41] Mazzariol S, Centelleghe C, Di Provvido A, Di Renzo L, Cardeti G, Cersini A, et al. Dolphin Morbillivirus Associated with a Mass Stranding of Sperm Whales, Italy. Emerg Infect Dis. 2017 Jan;23(1):144–6. doi: 10.3201/eid2301.160239 27983493 PMC5176224

[42] Pautasso A, Iulini B, Grattarola C, Giorda F, Goria M, Peletto S, et al. Novel dolphin morbillivirus (DMV) outbreak among Mediterranean striped dolphins *Stenella coeruleoalba* in Italian waters. Dis Aquat Organ. 2019;132(3):215–20. 31188137 10.3354/dao03323

[43] Soto S, Alba A, Ganges L, Vidal E, Raga J, Alegre F, et al. Post-epizootic chronic dolphin morbillivirus infection in Mediterranean striped dolphins *Stenella coeruleoalba*. Dis Aquat Organ. 2011 Oct 6;96(3):187–94. 22132497 10.3354/dao02387

[44] Díaz-Delgado J, Groch KR, Sierra E, Sacchini S, Zucca D, Quesada-Canales Ó, et al. Comparative histopathologic and viral immunohistochemical studies on CeMV infection among Western Mediterranean, Northeast-Central, and Southwestern Atlantic cetaceans. PloS One. 2019. 14(3):e0213363. doi: 10.1371/journal.pone.0213363 30893365 PMC6426187

[45] Cerutti F, Giorda F, Grattarola C, Mignone W, Beltramo C, Keck N, et al. Specific capture and whole-genome phylogeography of Dolphin morbillivirus. Sci Rep. 2020 Nov 30;10(1):20831. doi: 10.1038/s41598-020-77835-z 33257791 PMC7704663

[46] Wedekin LL, Neves MC, Marcondes MCC, Baracho C, Rossi-Santos MR, Engel MH, et al. Site fidelity and movements of humpback whales (*Megaptera novaeangliae*) on the Brazilian breeding ground, southwestern Atlantic. Mar Mammal Sci. 2010 Oct;26(4):787–802.

[47] Costa APB, Fruet PF, Secchi ER, Daura-Jorge FG, Simões-Lopes PC, Di Tullio JC, et al. Ecological divergence and speciation in common bottlenose dolphins in the western South Atlantic. J Evol Biol. 2021 Jan 27;34(1):16–32. doi: 10.1111/jeb.13575 31808214

[48] Rossi-Santos MR, Wedekin LL, Monteiro-Filho ELA. Residence and site fidelity of *Sotalia guianensis* in the Caravelas River Estuary, eastern Brazil. J Mar Biol Assoc United Kingdom. 2007 Feb 26;87(1):207–12.

[49] Sucunza F, Danilewicz D, Cremer M, Andriolo A, Zerbini AN. Refining estimates of availability bias to improve assessments of the conservation status of an endangered dolphin. Li S, editor. PLoS One. 2018 Mar 13;13(3):e0194213. doi: 10.1371/journal.pone.0194213 29534086 PMC5849330

[50] Audino T, Grattarola C, Centelleghe C, Peletto S, Giorda F, Florio C, et al. SARS-CoV-2, a Threat to Marine Mammals? A Study from Italian Seawaters. Animals. 2021 Jun 3;11(6):1663. doi: 10.3390/ani11061663 34204885 PMC8226612

[51] Mathavarajah S, Dellaire G. Lions, tigers and kittens too: ACE2 and susceptibility to COVID-19. Evol Med Public Heal. 2020 Jan 1;2020(1):109–13. doi: 10.1093/emph/eoaa021 32974030 PMC7337683

